# Hospital Costs of Colorectal Cancer Care

**DOI:** 10.4137/cmo.s2362

**Published:** 2009-03-20

**Authors:** D.A.L. Macafee, J. West, J.H. Scholefield, D.K. Whynes

**Affiliations:** 1 Division of GI Surgery, Queen’s Medical Centre, Nottingham.; 2 Division of Epidemiology and Public Health, University of Nottingham, Queen’s Medical Centre, Nottingham.; 3 School of Economics, University of Nottingham.

**Keywords:** colorectal cancer, cost, hospital, Dukes stage, screening

## Abstract

**Objective:**

In a hospital based setting, identify factors which influence the cost of colorectal cancer care?

**Design:**

Retrospective case note review

**Setting:**

Nottingham, United Kingdom

**Participants:**

227 patients treated for colorectal cancer

**Methods:**

Retrospective review of the hospital records provided the primary data for the costing study and included all CRC related resource consumption over the study period.

**Results:**

Of 700 people identified, 227 (32%) sets of hospital notes were reviewed. The median age of the study group was 70.3 (IQR 11.3) years and there were 128 (56%) males. At two years, there was a significant difference in costs between Dukes D cancers (£3641) and the other stages (£3776 Dukes A; £4921 Dukes B). Using univariate and multivariate regression, the year of diagnosis, Dukes stage of disease, intensive nursing care, stoma requirements and recurrent disease all significantly affected the total cost of care.

**Conclusions:**

CRC remains costly with no significant difference in costs if diagnosed before compared to after 1992. Very early and very late stage cancers remain the least costly stage of cancers to treat. Other significant effectors of hospital costs were the site of cancer (rectal), intensive nursing care, recurrent disease and the need for a stoma.

## Introduction

Colorectal cancer (CRC) care is costly partly because it is common (16000 deaths per year) and involves expensive, specialist hospital based operative intervention. In addition, its natural history of recurrence has prompted long periods in follow up programmes with additional operative intervention or palliative care for those with detected recurrences. This cost is set to rise further with the introduction of population based screening for CRC last year for 60 year olds in England, at a cost of £37.5 million in the first two years alone.^1^

Although costing for CRC is therefore important, there remains considerable variation in costing estimates, even if subdivided by stage of disease (Appendix 1).^2^–^9^ In addition, management of this disease has changed in many areas, with shorter hospital stays, increasing use and range of adjuvant therapies and calls for more intensive follow up.^10^,^11^ Whynes et al. examined the costs of hospital based care, publishing their findings in the BJC in 1993.^3^ We undertook a similar costing exercise to examine whether hospital costs have altered significantly, whether very early and very late stage cancers remain the least costly, and to consider the implications for changes in future management such as population based screening.

## Methods

### Study population

Following Trust Research and Development approval, people with histologically proven CRC diagnosed between February 1981 and August 2002 were identified from computerised pathology records of the audit department at Queen’s Medical Centre, Nottingham. 700 cases of CRC were identified. The list was ordered chronologically and every third set of notes was selected, with 227 cases being reviewed. Retrospective review of the hospital records provided the primary data for the costing study and included all CRC related resource consumption over the study period (e.g. initial assessment, operative intervention and outpatient follow up) (Appendix 2). Consumed resources related to research projects or community based activities (e.g. palliative care) were excluded. As the initial admission for treatment can underestimate the total hospital based costs, the costing period was defined as the time from primary operation or diagnosis to death, discharge from hospital based follow up or the study end date (01/09/2002). Many factors were analysed, with year of diagnosis being grouped as either pre or post 01/01/1992.

### Costs

A pragmatic approach to costing was taken using existing market costs rather than calculating opportunity cost. The year of operation was considered the base year (Year 0) with costs from later calendar years being discounted at 3%. Costs were in pounds sterling (£) and a treatment level perspective was taken for the analysis. The direct hospital costs, listed in Appendix 3, were obtained from three sources: the hospital finance department (personal communication Ms O’Connor), 2001 NHS reference costs^12^ and the Nottingham City Hospital pharmacy. Costs were extrapolated to 2001 costs, using the Gross Domestic Product deflator.^13^

### Statistical analysis

The basic demographics were described in terms of median values and their accompanying interquartile ranges (IQR), with non-parametric tests being used where appropriate (Chi square test for proportions, Kruskal Wallis H test) as the cost data was not normally distributed. Following log_e_ transformation of total and follow up costs, univariate and multivariate linear regression analysis was performed. The multivariate regression included all possible explanatory variables in the model as categorical variables with exponentiated coefficients and confidence intervals being used to calculate the geometric mean ratios. Statistical analysis was performed in SPSS 11.0 (SPSS Inc, Chicago) and Stata 7.0 (Stata Corporation, Texas).

## Results

Of 700 people identified, 227 (32%) sets of hospital notes were reviewed. Table 1 presents the basic demographics of the study group, by Dukes stage of disease. The median age of the study group was 70.3 (IQR 11.3) years and there were 128 (56%) males. The majority of cases were elective admissions (75%) although the proportion of emergency cases increased with advancing stage. At the study end date, 78 (34%) were still alive. More left sided cancers were resected in the early stages of disease and proportionally more stomas were formed in those with advanced disease. In 12 cases, the site of the tumour and its corresponding Dukes stage were unknown.

Patients with Dukes A cancers spent the least number of days in hospital, made up the highest proportion of the alive group and 45% (n = 14) had been discharged from follow up by the study end date. Their median age at diagnosis was 68.3 (IQR 11.0) years and their median time from operation to death or discharge was 7.7 (IQR 7.5) years, compared to 1.2 years (IQR 7.5) for Dukes D cancers.

Table 2 presents the two year and total cost by various demographic, disease and treatment characteristics that were considered. Across the whole group, the median two year cost was £4479 (IQR 4155) and the total cost was £5376 (IQR 5312). Within the first two years, there was no difference in cost by gender but overall, males cost £1186 more than females and survived one month longer. Subjects under 70 years of age survived 1.9 years longer than those over 70 although there was no statistically significant cost difference.

Known sites cost on average £3000 more than unknown sites (at two years) but the survival difference was marked (up to 6.5 years). Overall, rectal cancers cost £2793 more than a right sided cancer (caecum to splenic flexure) and the marked increase in costs related to intensive nursing care requirements were statistically significant at both the two year stage (£2350 *p* < 0.01) and overall. This is despite those patients having a marked shortening of their life expectancy (3.9 years). The requirement of a stoma also saw a statistically significant increase in cost over the two year and total period of follow up (*p* < 0.01). Recurrent disease was a predictor of increased costs, with a statistically significant increase in costs at two years (p = 0.01) and overall (p < 0.05).

Considering disease stage, unknown cancers cost the least (p < 0.01) and spent the least time in follow up. However, even when unknown cancers were excluded, a significant difference in two year costs was detected between Dukes D cancers (£3641) and the other stages (range £3776 Dukes A to £4921 Dukes B). This significance did not persist for total costs probably due to widening interquartile ranges.

When undertaking univariate analysis, the occurrence of rectal cancer, the need for a stoma, recurrent disease, or a Dukes B or C cancer predicted a significant difference in costs (Table 3). When all possible explanatory variables were submitted to multivariate regression, Dukes A to C, intensive nursing care, stoma requirements and recurrent disease all reached significance.

In order to take account of the wide variation in follow up periods, Table 4 considers the total cost per month by Dukes stage and various characteristics. Dukes C and particularly Dukes D cancers cost significantly more per month, with the significance becoming more prominent when unknown cancer stage was excluded. Late stage cancers cost £110 per month more but survived 2.5 years less than earlier stage cancers while the influence of intensive nursing care, recurrence or stoma costs on costs persisted. With multivariate regression, Dukes stage A and C, intensive nursing care, recurrent disease and year of diagnosis predicted a significant difference in costs.

## Discussion

### Findings

CRC remains costly with no significant difference in costs if diagnosed before compared to after 1992. Very early and very late stage cancers remain the least costly stage of cancers to treat. Other significant affectors of hospital costs were the site of cancer (rectal), intensive nursing care, recurrent disease and the need for a stoma.

### Strengths and limitations

#### The method, dataset and data capture

The decision to limit the study to around 200 sets of notes was considered a compromise between an adequate sample size and the limitations of time and costs. Ordering the dataset by hospital number and sampling every third set of notes was considered an appropriate randomisation method. Choosing the work of only one institution may limit the generalisability of results although there was no evidence that the clinical management in QMC differed from other U.K. centres. Hospital Episodes Statistics (HES) were not used as they were not a recognised research tool for the time period of the study (i.e. 1981 onwards) and they have no individual level data, so detailed costing techniques would have been sacrificed for the larger sample size.^14^,^15^ An initial inspection of the full data set would have been useful to look for trends in disease stage and other factors over time; however, this would have required a review of every set of notes which was not feasible in the time period.

What is recorded in hospital records is determined not by the needs of research but by what is felt relevant to direct patient care so the quality of this recording for research purposes was variable and sometimes incomplete. The cost of chemotherapeutic agents may have been underestimated as the majority of regimens included in this study were administered within trial settings under oncologists. Improved cost data is becoming available, looking at both adjuvant and palliative chemotherapy and also oral versus intravenous administration.^16^

#### Appropriate end points for costs

Following all patients until their death would have provided a more complete picture of the hospital based costs as death is a useful economic end point, where one can be certain of no further costs being incurred. However, in the study 66% of subjects had died by the end date of the study, with a further proportion (mainly Dukes A and B cancers) discharged from further hospital based follow up. The proportion of Dukes A cancers were small but corresponded with national figures, which found small proportions of very early or very late stage cancers (60.3% Dukes B or C; 15.4% unknown stage).^17^

#### Costs by stage of cancer and various characteristics

The inclusion of the twelve cases of unknown stage (which were all likely to be Dukes D cancers) proved to have a considerable effect on the results despite their short survival. Their stage was unknown because they either underwent palliative surgery (e.g. colostomy rather than tumour resection) or medical palliative care and none received intensive nursing care, palliative chemotherapy or any follow up. The main tables and multivariate analyses present data with unknown stage excluded but further analysis is presented beneath them, with all patients included. As they survived such a short period of time their costs are unsurprisingly very small.

Considering the remaining four Dukes stages, Dukes D cancers appeared to consistently incur the least hospital based costs. Of those with Dukes D cancer, 71% had a palliative or diagnostic operation with a similar post-operative stay to intermediate stage cancers. Although the lower costs could be partly related to less radical operative intervention, the most likely reason for the reduced costs is the short survival period post discharge. Outpatient costs were on average £1067 less than follow up of a Dukes A cancer.

Considering the European publications, the results are fairly similar to previous prospective studies by Whynes in 1993, although considerably less than the figures used for the economic modelling of the U.K. national screening trial (Appendix 1).^3^,^9^ With a statistically significant difference in costs between different stages of disease, it raises concerns over the use of a single NHS reference cost for operations on any cancer stage, as seen in the costing paper on flexible sigmoidoscopy screening in the U.K. Charitable donations are another reason for cost disparity as in the U.K. currently, most palliative care facilities require considerable charitable donations on top of limited direct NHS funds. The NHS cancer plan has budgeted for hospices and other facilities, so the economics in this area are likely to change considerably.^18^

Studies from Medicare patients in the United States found costs were four times higher for more advanced disease than for early stages yet the mean total cost of palliative care in the U.K. for any cancer was estimated to be only £2828 (1999).^19^,^20^ One of the main difficulties with U.S. economic data is that it is based on market costs and so rarely reflects the true cost of providing a service.

Despite a lower risk of recurrence, more was spent on outpatient visits and investigations for Dukes A cancers than for either Dukes B or C cancers. Considering areas for potential cost saving, targeted follow up of very early cancers could be one area for consideration.^21^

Rectal cancers appeared to be the most costly site of cancer, possibly due to an increased risk of local recurrence or the need for a stoma (temporary or permanent).

There is currently little published work on the daily resource use of stoma patients despite stoma care products being essential, costly and commonly prescribed. The high daily resource use (two bags per day plus condiments) and the cost of each bag (£2.2) probably explains why having a stoma effected overall costs so significantly, particularly if the stoma was permanent. Further costs were incurred due to operations for the complications or reversal of such stomas.

### Implications

Population based screening in England raises additional CRC cost issues. Previous studies have suggested that significant cost savings should not be expected from population screening initially despite the increased number of early cancers detected.^22^ Setting up such a programme is costly before any subject is actually screened and it is important that health planners have ensured that resources are available both in terms of investigations (e.g. endoscopy, radiology) and colorectal services (e.g. medical and nursing staff).

Further developments of chemotherapeutic agents and completion of the trials proving their clinical effectiveness may intensify the management of advanced stage disease. A recent study estimated palliative chemotherapy to cost between £2576 and £5051, depending on the therapy used.^23^ The use of oral chemotherapeutic agents may reduce the inpatient costs of such treatments but we may ultimately see a similar trend to hospital costs as in the U.S, with advanced stage disease costing as much or more than early, curable disease despite shorter survival periods.

Payment by results and the use of National Tariffs has meant that hospitals receive a set cost from primary care trusts for certain operative procedures (National Tariffs) irrespective of how costly it is to provide the service.^24^ Whether this will encourage individual trusts to analyse their costs more closely (and so improve health economic data in this area) is unclear. NHS reference costs remain the most widely used and accepted data in the NHS to date.

Hospital based colorectal cancer care is costly. Population screening, laparoscopic colorectal surgery, intensive follow up and polyp surveillance, new chemotherapeutic agents and more aggressive surgery for metastatic disease are likely to see these costs rise further. This may well be partially offset by the identification of early stage polyps and cancers, fewer stomas, less need for intensive care (less invasive laparoscopic surgery), targeted follow up programmes and shorter hospital stays but individual trusts may have to scrutinise their costs more closely if the National Tariffs do not adequately reward them for the care provided. Overall, all the above should hopefully see a fall in CRC mortality and better patient care despite the costs.

## Figures and Tables

**Table 1.** Characteristics of the study group by Dukes stage.

**Characteristics**		**Dukes stage of disease**				
		**A (n = 31)**	**B (n = 76)**	**C (n = 77)**	**D (n = 31)**	**Unknown (n = 12)**
Male	n (%)	20 (65)	43 (57)	44 (57)	15 (48)	6 (50)
Age at operation in years	Median (IQR)	68.3 (11.0)	71.6 (10.9)	71.1 (10.8)	68.8 (9.8)	71.6 (27.3)
Elective operation	n (%)	30 (97)	67 (88)	52 (68)	22 (71)	0
Rectal cancers	n (%)	19 (61)	28 (37)	33 (43)	11 (36)	–
Stoma formed	n (%)	9 (29)	15 (20)	30 (40)	15 (48)	1 (17)
Intensive nursing required	n (%)	1 (3)	8 (11)	4 (5)	0	0
Number of inpatient days	Median (IQR)	9.0 (5.7)	14.0 (8.0)	12.0 (7.5)	13.0 (10.0)	15.0 (36.5)
Recurrent disease at some time	n (%)	9 (29)	17 (22)	45 (58)	–	–
Chemotherapy required at some time	n (%)	1 (3)	6 (8)	20 (26)	3 (10)	0
Alive at end date	n (%)	19 (61)	34 (45)	25 (33)	0	0
Discharged from follow up	n (%)	14 (45)	27 (36)	21 (27)	3 (10)	0
Time from operation to death in years	Median (IQR)	7.7 (7.5)	6.2 (7.9)	4.9 (6.9)	1.2 (7.5)	0 (0.6)
Diagnosis before year 1992	n (%)	13 (42)	25 (33)	20 (26)	11 (35)	5 (42)
Colorectal cancer related death	n (%)	3 (10)	13 (17)	24 (31)	23 (74)	7 (58)

**Table 2.** Cost at two year follow up and total costs by Dukes stage and various characteristics.

**Characteristics**	**Number n (%)**	**Median cost at two years £ (IQR)**	**p value**	**Median total cost £ (IQR)**	**p value**	**Median time in follow up program years (IQR)**
**Stage of disease**						
Dukes A	31 (14)	3776 (3280)		4936 (6670)		7.7 (7.5)
Dukes B	76 (34)	4670 (3954)		6388 (5710)		6.2 (7.9)
Dukes C	77 (34)	4921 (3779)		5673 (4782)		4.9 (6.9)
Dukes D	31 (14)	3641 (5937)		3641 (6207)		1.2 (7.4)
Unknown	12 (5)	1669 (2571)	p < 0.05*	1818 (5547)	p = 0.06*	0 (0.6)
Male	128 (56)	4643 (3989)		6000 (6012)		5.4 (10.7)
Female	99 (44)	4184 (3963)	p = 0.06	4814 (5340)	p < 0.05	4.4 (7.4)
**Site of cancer**						
Rectum	92 (41)	4947 (4507)		7189 (4504)		6.5 (11.0)
Left sided	67 (30)	4333 (4003)		5355 (6117)		5.9 (7.8)
Right sided	60 (26)	4180 (3097)		4396 (3986)		2.2 (7.1)
Unknown	8 (4)	1048 (2164)	p < 0.05**	1197 (2164)	p < 0.05**	0 (0.6)
Standard nursing care	214 (94)	4391 (3784)		5323 (5426)		5.4 (8.4)
Intensive nursing care	13 (6)	6741 (6709)	p < 0.01	7670 (7175)	p < 0.05	1.5 (6.8)
No stoma	157 (69)	4185 (2749)		4750 (4956)		5.1 (8.4)
Stoma formed	70 (31)	5663 (4391)	p < 0.01	7701 (5656)	p < 0.01	5.4 (6.9)
No recurrence	103 (45)	4206 (2940)		4868 (4567)		7.3 (11.2)
Recurrence	124 (55)	4930 (5268)	p = 0.01	6459 (7921)	p < 0.05	2.4 (5.6)
Pre 1992	74 (33)	4466 (3520)		6070 (5516)		13.1 (10.8)
1992 onwards	153 (67)	4479 (4288)	p = 0.80	5159 (5336)	p = 0.18	2.7 (6.1)

Costs in Pounds Sterling (2001), discounted at 3% rate. Median costs (Interquartile range).

* When unknown stage included, median cost at two years p < 0.01; median total cost p < 0.01.

** When unknown site included, median cost at two years p < 0.01; median total cost p < 0.01.

**Table 3.** Univariate analysis and multivariate regression of total costs in all patients.

**Dependent variable**	**Ratio of geometric mean (95% confidence interval)**			
	**Univariate**	**Significance (p)**	Multivariate**	**Significance (p)**
**Stage of cancer**				
Dukes A	1.2 (0.9–1.6)	0.27	1.5 (1.1–2.0)	<0.05
Dukes B	1.4 (1.0–1.8)	<0.05	1.8 (1.4–2.4)	<0.01
Dukes C	1.4 (1.1–1.8)	<0.05	1.5 (1.2–2.0)	<0.01
Dukes D*	1	–	1	
Male	1.2 (1.0–1.4)	0.05	1.1 (0.9–1.3)	0.28
Female*	1		1	
**Site of cancer**				
Rectum	1.3 (1.1–1.6)	<0.05	1.1 (0.9–1.4)	0.29
Left sided	1.1 (0.9–1.4)	0.47	1.0 (0.8–1.3)	0.91
Right sided*	1		1	
Standard nursing care	0.7 (0.5–1.0)	0.06	0.7 (0.5–0.9)	<0.05
Intensive nursing care*	1		1	
No stoma	0.8 (0.6–0.9)	<0.01	0.8 (0.6–0.9)	<0.01
Stoma formed*	1		1	
No recurrence*	1		1	
Recurrence	1.3 (1.1–1.5)	<0.01	1.5 (1.2–1.8)	<0.01
Pre 1992	1.1 (1.0–1.4)	0.13	1.2 (1.0–1.4)	<0.05
1992 onwards*	1		1	

* Baseline category. Signficance (p)—0.05 level.

** Customised multivariate linear regression.

If unknown stage of cancer included in univariate analysis, ratio of geometric means were Dukes A 4.5 (p < 0.01); Dukes B 5.1 (p < 0.01); Dukes C 5.2 (p < 0.01); Dukes D 3.8 (p < 0.01) If unknown stage of cancer included in multivariate regression, ratio of geometric means were Dukes A 2.2 (p < 0.05); Dukes B 2.7 (p < 0.01); Dukes C 2.3 (p < 0.05); Dukes D 1.5 (p = 0.24).

**Table 4.** Total cost per month in all cases, by Dukes stage and various characteristics.

**Characteristics**	**Number n (%)**	**Median cost per month post diagnosis £ (IQR)**	**p value**	**Median time in follow up program years (IQR)**
**Stage of cancer**				
Dukes A	31 (14)	69 (105)		7.7 (7.5)
Dukes B	76 (34)	104 (299)		6.2 (7.9)
Dukes C	77 (34)	151 (426)		4.9 (6.9)
Dukes D	31 (14)	370 (1197)		1.2 (7.4)
Unknown	12 (5)	81 (−)	p < 0.01*	0 (0.6)
Male	128 (56)	112 (367)		5.4 (10.7)
Female	99 (44)	116 (317)	p = 0.82	4.4 (7.4)
**Site of cancer**				
Rectum	92 (41)	94 (321)		6.5 (11.0)
Left sided	67 (30)	118 (253)		5.9 (7.8)
Right sided	60 (26)	196 (528)		2.2 (7.1)
Unknown**	8 (4)	0	p = 0.18	0 (0.6)
Standard nursing care	214 (94)	104 (327)		5.4 (8.4)
Intensive nursing care	13 (6)	428 (9580)	p < 0.01	1.5 (6.8)
No stoma	157 (69)	91 (333)		5.1 (8.4)
Stoma formed	70 (31)	205 (428)	p < 0.05	5.4 (6.9)
No recurrence	103 (45)	75 (146)		7.3(11.2)
Recurrence	124 (55)	256 (506)	p < 0.01	2.4 (5.6)
Pre 1992	74 (33)	46 (91)		13.1 (10.8)
1992 onwards	153 (67)	174 (437)	p < 0.01	2.7 (6.1)

Costs in Pounds Sterling (2001), discounted at 3% rate. Median costs (Interquartile range).

* When unknown stage included, p < 0.05; when excluded p < 0.01.

** Unknown site excluded as costs per month negligible and rejected by SPSS.

## Abbreviations

CRC: Colorectal cancer;
Dukes: Dukes stage of disease;
NHS: National Health Service.

## Appendix

**Appendix 1.** Published hospital based costs of symptomatic colorectal cancer care by cancer stage.

**Author Year Currency Journal/Report Unit cost or charge Costs included Type of study**	**Tuck^2^ UK Sterling (£) Journal Unit Hospital costs Prospective**	**Whynes^3^ UK Sterling (£) Journal Unit Hospital costs Prospective**	**Loeve^4^ US Dollars ($) Journal Charge Hospital Microsimulation**	**Frazier^5^ US Dollars ($) Journal Charge Lifetime Microsimulation**	**Ramsey^6^ US Dollars ($) Journal Charge 1 yr Hospital Database review**	**Selke^7^ Journal Charge/Unit Hospital Database review**	**Whynes^3^ UK Sterling (£) Journal Unit Hospital costs NHS reference costs**	**Alexander^9^ UK Sterling (£) Report Unit cost Lifetime Markov model**
**Dukes’ stage**						HDG cost	HDG cost	
A (£)	Average cost	3421	Average cost	12190	13057	Average cost	Average cost	6825
B (£)	for all stages	4373	for all stages	12190	18341	for all stages	for all stages	7154
C (£)	2834	4461	15157	24313	18038	2370	3504	6922
D (£)		3402		32288	16205			5786

All prices updated to 2001 prices, and converted to U.K. pounds sterling (£1 = $1.91 = €1.44).

## Appendix

**Appendix 2.** Information extracted from the hospital records.

**Section 1: Patient demographics**
**Section 2: Initial operation and post operative care**
Pre-operative investigations
Operation type and post operative management and morbidity
Histopathological results—Dukes stage of disease
**Section 3: Hospital based follow up**
Timing and indication for outpatient appointment
Clinic, endoscopy and radiology based investigations (e.g. Ultrasound, Colonoscopy)
**Section 4: Adjuvant therapy and stoma care**
Type of adjuvant therapy and associated morbidity
Type of stoma
**Section 5: Further operations (e.g. recurrent disease, metachronous lesions)**
As for initial operation
**Section 6: Medical comorbidity and mortality**
Medical comorbidity (e.g. Myocardial infarction)
Cause and date of death (post mortem findings if available)

## Appendix

**Appendix 3.** Various “direct” hospital costs and their origin.

**Setting**	**Hospital resource**	**Cost (£) pounds sterling**	**HRG code***
**Inpatient**			
Operation	Major operation	£2,494	F34
	Minor operation (e.g. reversal of stoma)	£780	F44
Post operative care	Adult intensive care unit	£1263/day	QMC+
	Surgical High dependency unit	£99 1/day	QMC+
	Surgical ward	£370/day	QMC+
**Outpatient**			
Surgical clinic	New patient appointment	£93	QMC+
	Follow up appointment	£47	QMC+
Endoscopy	Flexible sigmoidoscopy	£119	F14op
	Colonoscopy	£127	F06op
Radiology	Chest X ray	£71	F20op
	Ultrasound of liver	£91	F18op
	Double contrast barium enema	£120	F15op
	CT abdomen/pelvis	£143	F04op
	MRI pelvis	£188	F03op
Adjuvant therapy	Pre or post operative radiotherapy	£500	NCH*
	Chemotherapy	£709–8322	NCH*

HRG code—NHS reference costs 2001.

Queens Medical Centre QMC+ submissions for NHS reference costs Chemotherapy costs NCH* from Nottingham City Hospital Pharmacy depended on the treatment regimen (Quasar 5/7 trial £709 to Irinotecan/5FU/FA £8322).
